# Mepivacaine reduces calcium transients in isolated murine ventricular cardiomyocytes

**DOI:** 10.1186/s12871-019-0926-0

**Published:** 2020-01-08

**Authors:** Matias Mosqueira, Güçlü Aykut, Rainer H. A. Fink

**Affiliations:** Cardio-Ventilatory Muscle Physiology Laboratory, Institute of Physiology and Pathophysiology, Heidelberg University Hospital, Im Neuenheimer Feld 326, 69120 Heidelberg, Germany

**Keywords:** Local anesthetic, Mepivacaine, Ca^2+^ transients, Mouse, Cardiomyocytes

## Abstract

**Background:**

The potential mechanism of mepivacaine’s myocardial depressant effect observed in papillary muscle has not yet been investigated at cellular level. Therefore, we evaluated mepivacaine’s effects on Ca^2+^ transient in isolated adult mouse cardiomyocytes.

**Methods:**

Single ventricular myocytes were enzymatically isolated from wild-type C57Bl/6 mice and loaded with 10 μM fluorescent Ca^2+^ indicator Fluo-4-AM to record intracellular Ca^2+^ transients upon electrical stimulation. The mepivacaine effects at half-maximal inhibitory concentration (IC_50_) was determined on calibrated cardiomyocytes’ Ca^2+^ transients by non-parametric statistical analyses on biophysical parameters. Combination of mepivacaine with NCX blockers ORM-10103 or NiCl_2_ were used to test a possible mechanism to explain mepivacaine-induced Ca^2+^ transients’ reduction.

**Results:**

A significant inhibition at mepivacaine’s IC_50_ (50 μM) on Ca^2+^ transients was measured in biophysical parameters such as peak (control: 528.6 **±** 73.61 nM vs mepivacaine: 130.9 **±** 15.63 nM; *p* < 0.05), peak area (control: 401.7 **±** 63.09 nM*s vs mepivacaine: 72.14 **±** 10.46 nM*s; *p* < 0.05), slope (control: 7699 **±** 1110 nM/s vs mepivacaine: 1686 **±** 226.6 nM/s; *p* < 0.05), time to peak (control: 107.9 **±** 8.967 ms vs mepivacaine: 83.61 **±** 7.650 ms; p < 0.05) and D_50_ (control: 457.1 **±** 47.16 ms vs mepivacaine: 284.5 **±** 22.71 ms; p < 0.05). Combination of mepivacaine with NCX blockers ORM-10103 or NiCl_2_ showed a significant increase in the baseline of [Ca^2+^] and arrhythmic activity upon electrical stimulation.

**Conclusion:**

At cellular level, mepivacaine blocks Na^+^ channels, enhancing the reverse mode activity of NCX, leading to a significant reduction of Ca^2+^ transients. These results suggest a new mechanism for the mepivacaine-reduction contractility effect.

## Background

Hemodynamic changes caused by local anesthetics may be of major clinical concern. Among others, these hemodynamic effects associated with local anesthetics include a decrease in ventricular conduction velocity leading to arrhythmias and a decrease in myocardial contractility [1–3]. Mepivacaine is a widely used amide-type local anesthetic, which demonstrates a rapid metabolism via the liver and a rapid excretion via the kidneys. Clinically, mepivacaine shows a short onset time, intermediate duration and low toxicity, which can therefore be regarded as a safe drug in high risk cardiac patients [4]. However, it has been shown that mepivacaine induces shortening of the QRS complexes, decreases of QTc-interval and ventricular arrhythmias [5, 6]. In dogs, mepivacaine induces decrease of the heart rate, cardiac output and stroke volume, increasing the area-under-curve of T-wave, lengthening QTU interval. These cardiac alterations can be explained as 40 μM mepivacaine-induced reduction in the Na^+^ current’s (I_Na_) measured parameters such as overshoot, dv/dt_max_ and action potential at 20% of repolarization (APD_20_) [7]. As consequence of I_Na_ reduction, myocardial depression in ventricular papillary muscles caused by mepivacaine has also been reported [8, 9], without the potential mechanism of mepivacaine’s myocardial depressant effect. Additionally, to our knowledge there are no conclusive studies about direct effects of mepivacaine at cellular level, which would clarify the mechanism of mepivacaine’s effect on intrinsic myocardial contractility. Therefore, we hypothesized that known mepivacaine’s effect on I_Na_ might cause a reduction of Ca^2+^ transient at the IC_50_ for isolated murine cardiomyocyte, explaining the negative inotropic effect of mepivacaine previously described. To test this hypothesis we analyzed Ca^2+^ transients’ biophysical parameters established in our laboratory [10–12]. We observed in isolated adult mouse ventricular cardiomyocytes that mepivacaine’s effect at the IC_50_ calculated from the dose-response analysis, which is considered supra-clinical in humans, significantly reduced Ca^2+^ release from the sarcoplasmic reticulum. One suggested mechanism to explain this reduction on Ca^2+^ transient is the NCX reverse mode activity, that would reduce [Ca^2+^]_i_ in order to keep the [Na^+^]_i_. The final result supports this hypothesis showing that the combination of mepivacaine and an specific NCX blocker ORM10103 or NiCl_2_ alters the Ca^2+^ homeostasis.

## Methods

### Ethical statement

All animal experiments were performed in accordance with the governmental guidelines of the state Baden Württemberg, including the German law on animal experimentation, and were approved by the ethics committee of Heidelberg University Interfaculty Biomedical Research Facility (T84/14) and followed relevant aspects of ARRIVE guidelines. Isolated cardiomyocytes were obtained from five 6-month male C57 BL/6 NCrl mice purchased from Charles River, Europe and kept in the Interfaculty Biomedical Research Facility until use under 12 h light/ dark cycle with food and water ad libitum.

### Cardiomyocyte isolation

Isolation of single cardiac cells was carried out in a modified Langendorff system by the method of Liao and Jain as described previously [13] and adapted by us [12]. Prior to dissection and cannulation of the heart, the perfusion system was filled with perfusion solution and cleared off any air bubbles. Twenty minutes after i.p. heparinization (8 mg) the mouse was killed by cervical dislocation. The heart was excised and quickly perfused using the Langendorff apparatus with a Ca^2+^ free perfusion solution for 5 min containing the following (in mM): NaCl: 135; KCL: 4; MgCl_2_*6H_2_O: 1; HEPES: 10; NaH_2_PO_4_: 0.33; Glucose: 10; BDM: 20; Taurine: 5 with a pH value of 7.2 at room temperature. Following the perfusion solution, a Ca^2+^ free digestion solution was applied which was consisting of Collagenase D 0,36 mg/g (Roche cat no. 11088858001), Collagenase B 0,48 mg/g (Roche cat no. 11088807001) and protease XIV: 0,06 mg/g (Sigma-Aldrich cat no. P5147) dissolved in 30 ml perfusion solution. After 20 min appropriate digestion, the heart was once more perfused with perfusion solution for 5 min to wash out the enzymes. The ventricles were then mechanically dissociated in Transfer Buffer-A (TB-A) solution containing (in mM): NaCl: 135; KCL: 4; MgCl_2_*6H_2_O: 1; HEPES: 10; NaH_2_PO_4_: 0.33; Glucose: 5.5; BDM: 15; BSA: 5 mg/ml; pH: 7.2 at room temperature. For plating, the cardiomyocytes were placed on 35 mm petri dishes (Imaging Dish CG cat no 5160–30, Zell-Kontakt GmbH, Germany) previously coated with extracellular matrix (ECM, cat no. E127, Sigma Aldrich, Germany). Extracellular Ca^2+^ content was increased in steps (from 0 to 0.6 and then to 1,2 mM) by mixing TB-A with a phosphate-free HEPES buffered Transfer Buffer-B (TB-B) solution containing the following (in mM): NaCl: 137; KCL: 5.4; MgCl_2_*6H_2_O: 1; CaCl_2_*2H_2_O: 1.8; HEPES: 10; Glucose: 5.5; pH: 7.4 at room temperature. Isolated myocytes were then incubated in TB-B containing 10 μM Fluo-4-acetoxymethyl ester (Fluo-4 AM), at 37 °C for 30 min until introduced into the experimental chamber.

### Fluorescence measurements

As previously described [12], the experimental chamber was mounted on an inverted microscope (Olympus OSP-3 photomultiplier system, Olympus, Tokyo, Japan). After the incubation with Fluo-4 AM, every petri dish was positioned on the experimental chamber and was filled TB-B solution. After an equilibration period with TB-B for 5 min, an appropriate myocyte was chosen for the measurement under the 20X objective with normal light source according to the following criteria: (a) rod-shaped appearance with clear striations and no membrane blebs, (b) a negative staircase of twitch performance on stimulation from rest, and (c) the absence of spontaneous contractions. Cardiomyocytes’ Ca^2+^ transients were examined with a fluorescence light system (Olympus) through a 40X objective (Olympus) using the 488-nm filter for a 75 W Xenon light source. Fluorescence emission was monitored at 510 nm. During recordings the cells were flanked with two platinum electrodes and field electrically stimulated (single twitch at 0.5 Hz, 25 V, 10 ms duration) using SI-Heidelberg Stimulator (Scientific Instruments-Heidelberg, Heidelberg, Germany). The signals were digitally recorded using PowerLab 4/35 (AD Instruments) and analyzed offline after calibration utilizing LabChart 8 software. For each cardiomyocyte ten consecutive stimulations were recorded, but only the last seven Ca^2+^ transients were averaged and analyzed in order to avoid differences in the sarcoplasmic reticulum’s Ca^2+^ loading due to inactivation between two recordings [14–16]. After control cells were examined in every dish, mepivacaine dissolved in TB-B solution as described in experimental protocol was equilibrated for 5 min before recording Ca^2+^ transients.

### Calibration

Calibration procedure for converting the voltage signal to Ca^2+^ concentration was performed at the end of every experiment as previously described [10–12]. Briefly, the background of the petri dish was recorded in 10 different spots (no cells). After incubation with calibration solution I containing (in mM): NaCl: 140; KCl: 5; KH_2_PO_4_: 1.2; MgCl_2_*6H_2_O: 1.2; CaCl_2_*2H_2_O: 4; HEPES: 20; Ionomycin: 0.005; CPA: 0.01; Caffeine: 5; Ouabain: 1 at room temperature, 10 cardiomyocytes incubated in 10 μM Fluo-4 AM at 37 °C for 30 min cells were recorded as maximum fluorescence (F_max_). Then the petri dish was washed twice with TB-B and incubated at room temperature with calibration solution II containing (in mM): LiCl: 140; KCl: 5; KH_2_PO_4_: 1.2; MgCl_2_*6H_2_O: 1.2; HEPES: 20; EGTA: 4; Ionomycin: 0.005; CPA: 0.01; Caffeine: 5; Ouabain: 1. At the end 10 cells were recorded as minimum fluorescence (F_min_). The range of Ca^2+^ concentration to established F_max_ and F_min_ was 0 to 4 mM, respectively. Once both F_max_ and F_min_ were determined, the background was subtracted and then used to transform voltage into Ca^2+^ concentration using the following formula: [Ca^2+^]_i_ = Kd [(F - F_min_)/ (F_max_ - F)] [17]. Kd is the apparent dissociation constant of the Fluo-4-AM provided by the manufacturer (345 nM) and F represents the Ca^2+^ transient signal in volts recorded directly from the photomultiplier. After the conversion from volt signal to Ca^2+^ concentration, the data were analyzed, as previously described.

### Analysis of Ca^2+^ transients

To determine the effect of mepivacaine on Ca^2+^ handling properties of the cells, biophysical parameters were analyzed in Peak Analysis extension from LabChart 8 software as previously mentioned [10–12]: baseline (Ca^2+^ concentration before the electrical stimulation; nM), peak area (area under the curve of the fluorescence signal; nM*s), duration (time from the initial until the end of Ca^2+^ transient; ms), duration_50_ (D_50_; duration of the transient at 50% of the maximal peak; ms), peak (increase of the signal from baseline to maximum fluorescence intensity; nM), slope (rapidity of increment of the fluorescence from baseline to maximum peak; nM/s), time to peak (time of increment of the fluorescence from baseline to maximum peak; ms), tau (time constant decay between 90 and 5% of the fluorescence signal), and time to uptake (time of decrement of the fluorescence from maximum peak to baseline; ms). These parameters were divided into three categories: the parameters which describe the general aspects of the Ca^2+^ transients (peak area, duration and D_50_), the parameters which evaluate the Ca^2+^ release (peak, time to peak and slope) and the parameters which specify the Ca^2+^ uptake (tau and time to uptake). All raw data were published in public archive (DOI: 10.6084/m9.figshare.7713074.v1).

### Experimental protocol

Concentration-response relationship of mepivacaine was investigated in order to calculate the inhibitory effect at 50% of the response (IC_50_) of mepivacaine for our experiments. Myocytes were exposed to six concentrations of mepivacaine (10, 20, 40, 50, 75 and 100 μM). Four 35 mm dishes were prepared from each heart to test three different concentration. First 5–10 myocytes on every dish were examined as controls with TB-B solution. Then TB-B was removed and the TB-B solution containing each concentration of mepivacaine. After the IC_50_ was determined, Ca^2+^ transients from 42 cardiomyocytes were analyzed in control solution. After a 5-min equilibration period with 50-μM mepivacaine, Ca^2+^ transients from 29 cardiomyocytes were recorded. For NCX contribution, 5–10 isolated cardiomyocytes in control solution were recorded from each plate, then 31 different isolated cardiomyocytes incubated with NiCl_2_ 1 mM or 24 isolated cardiomyocytes incubated ORM-10103 10 μM and then 10 cardiomyocytes recorded with the combination of NiCl_2_ and mepivacaine and 10 cardiomyocytes recorded with ORM and mepivacaine.

### Materials

Except where noted above, all reagents were obtained from Sigma Aldrich (Sigma Aldrich, Germany). All solutions (perfusion, digestion, TB-A, TB-B, calibration solution I and II) were prepared as stock solutions by using distilled water. After the pH was titrated, these stock solutions were diluted with distilled water to the appropriate concentration before use.

Mepivacaine hydrochloride (SML1444) was diluted in TB-B solution to obtain drug concentrations of 10, 20, 40, 50, 75 and 100 μM. For intracellular Ca^2+^ concentration measurements, Fluo-4 AM was obtained from Molecular Probes (F14201). Stock solutions of 1 mM Fluo-4 AM were prepared in DMSO (Hibri-Max, cat no. D2650,), stored at − 20 °C and dissolved in TB-B at 10 μM prior incubation. NiCl_2_ (451193-5G) and ORM-10103 (SML0972) were freshly dissolved in TB-B at 1 mM and 10 μM respectively.

### Statistical analysis

The power analysis was performed with G*Power software (G*Power 3.1.7 by Axel Buchner, University of Düsseldorf, Germany). Statistical analyses were performed using GraphPad Prism software, Version 7.0 for Windows (GraphPad Software, San Diego, CA, USA). To obtain IC_50_ of mepivacaine peak area (nM*s) log dose vs response analysis was used. Data were fitted by logistic regression and the IC_50_ value was calculated. As the biophysical parameter values of Ca^2+^ transient analyses did not show a normal distribution (Kolmogorov-Smirnov test), a two-tail nonparametric test (Mann-Whitney U) was used to compare the parameters between control and mepivacaine treated cardiomyocytes in the second protocol of the study. Since it was averaged 7 out of 10 Ca^2+^ transients from each recorded cardiomyocyte, the data are presented as the mean and standard error. The comparison of baseline between control, NCX blockers NiCl_2_ 1 mM, ORM-10103 10 μM and the combination of mepivacaine 50 μM and NCX blockers were performed using ANOVA one-way and Dunnett’s post-doc multiple comparison test. The data is presented as mean ± SEM and the differences were considered statistically significant at *p* < 0.05.

## Results

**Fig. 1:**
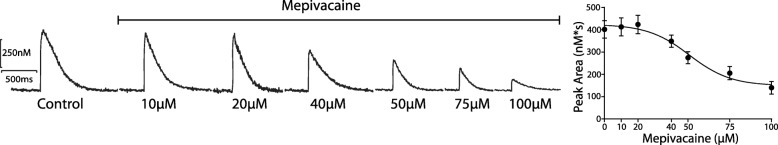
Representative Ca^2+^ transients’ traces to the dose-response effect of mepivacaine at 0, 10, 20, 40, 50, 75 and 100 μM (**a**). Log dose-response analysis of the peak area (nM*s) parameter was analyzed, obtaining the IC_50_ value of 50.85 ± 6.79 μM

To determine the IC_50_, the response as the biophysical parameter Peak Area (nM*s) obtained from the Ca^2+^ transients to log-dose concentrations of mepivacaine 0, 10, 20, 40, 50, 75 and 100 μM (Fig. 1a) was analyzed (Fig. 1b). The estimated IC_50_ value for mepivacaine was 50.85 **±** 6.79 μM. Therefore, and for the next protocol, the concentration of mepivacaine used was 50 μM and referred as mepivacaine alone.

**Fig. 2:**
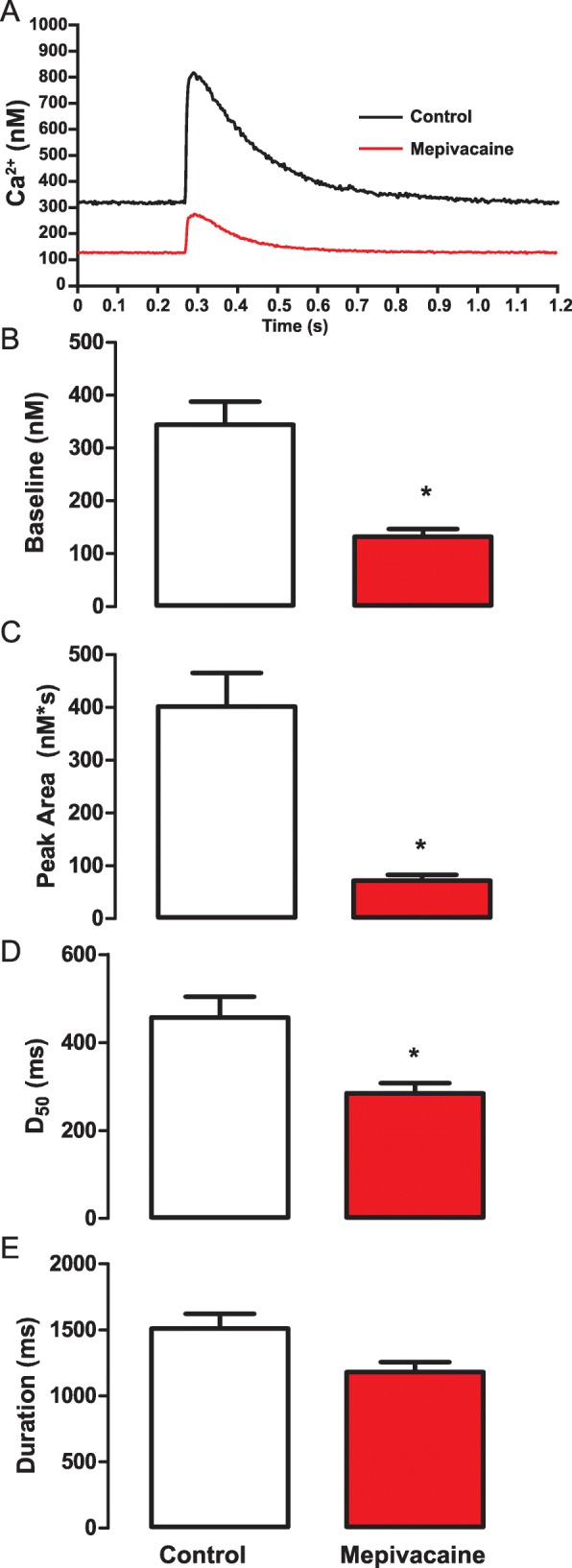
Representative Ca^2+^ transients trace demonstrating the inhibitory effect of mepivacaine at half maximal inhibitory concentration (IC_50_) on in isolated mouse ventricular cardiomyocytes (**a**). Summarized data for the effect of mepivacaine (IC_50_) on biophysical parameters describing the general aspects of the Ca^2+^ transients: baseline (**b**) peak area (**c**), Duration at 50% of the maximum D_50_ (**d**) and duration (**e**). Results are expressed as mean ± SEM. Symbol (*) indicates significant change from control (*p* < 0.05). *n* = 42 cells for control and *n* = 29 cells for mepivacaine group

**Fig. 3:**
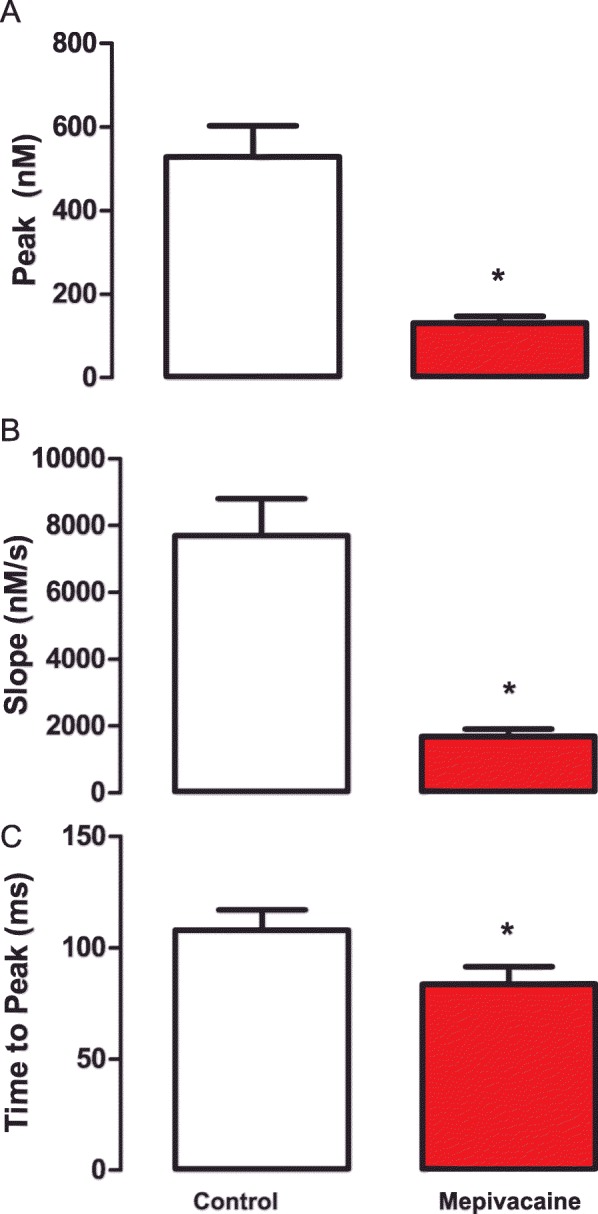
Summarized data for the effect of mepivacaine at IC_50_ on Ca^2+^ release’s biophysical parameters obtained from Ca^2+^ transients: peak (**a**), slope (**b**) and time to peak (**c**). Results are expressed as mean ± SEM. Symbol (*) indicates significant change from control (p < 0.05). n = 42 cells for control and n = 29 cells for mepivacaine group

The incubation of cardiomyocytes with 50 μM (IC_50_) of mepivacaine induced a drastic reduction of the Ca^2+^ transient compared to control (Fig. 2a). This reduction was confirmed by the no-parametric statistical analyses showing significant inhibition in several biophysical parameters of Ca^2+^ transients. Mepivacaine, significantly reduced the parameters that describe the general aspects of the Ca^2+^ transients, such as baseline (control: 356.8 **±** 42.12 nM; *n* = 42 cells vs mepivacaine: 132.2 **±** 14.55 nM; *n* = 29 cells; *p* < 0.05; Fig. 2b), peak area (control: 401.7 **±** 63.09 nM*s vs mepivacaine: 72.14 **±** 10.46 nM*s; *p* < 0.05; Fig. 2c) and D_50_ (control: 457.1 **±** 47.16 ms vs mepivacaine: 284.5 **±** 22.71 ms; *p* < 0.05; Fig. 2d). However, the total duration of the Ca^2+^ transient was not significantly reduced by mepivacaine (control: 1511 **±** 111.6 ms vs mepivacaine: 1181 **±** 76.46 ms; *p* > 0.05; Fig. 2e). Additionally, the biophysical parameters which evaluate the Ca^2+^ release showed that peak (control: 528.6 **±** 73.61 nM vs mepivacaine: 130.9 **±** 15.63 nM; p < 0.05; Fig. 3a), slope (control: 7699 **±** 1110 nM/s vs mepivacaine: 1686 **±** 226.6 nM/s; p < 0.05; Fig. 3b) and time to peak (control: 107.9 **±** 8.967 ms vs mepivacaine: 83.61 **±** 7.650 ms; p < 0.05; Fig. 3c) were significantly reduced under mepivacaine influence. Regarding the re-uptake biophysical parameters, mepivacaine had no significant effect such as tau (control: 0.7540 **±** 0.05374 vs mepivacaine: 0.6838 **±** 0.05698; *p* > 0.05), and time to uptake (control: 1405 **±** 112.1 ms vs mepivacaine: 1079 **±** 77.92 ms; p > 0.05).

**Fig. 4:**
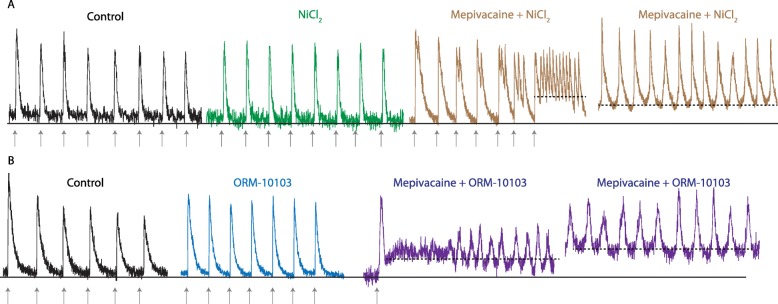
Representative Ca^2+^ transient traces from isolated murine cardiomyocyte under the effect of NCX blockers and in combination with mepivacaine. (**a**) Response of Ca^2+^ transient under 1 mM NiCl_2_ (green) did not significantly altered both baseline (solid black line) neither peak area parameters. Gray arrows indicate electrical stimulation. The combination of 50 μM mepivacaine with 1 mM NiCl_2_ (brown) induced arrhythmic activity and significant increase in the baseline (*p* < 0.01; dashed black line). *n* = 27 cells for control, 31 cells for NiCl_2_ 1 mM and 10 cells for Mepivacaine 50 μM + NiCl_2_ 1 mM. (**b**) Response of Ca^2+^ transient under 10 μM ORM-10103 (blue) did not significantly altered both baseline (solid black line) neither peak area parameters. The combination of 50 μM mepivacaine with 10 μM ORM-10103 (purple) induced arrhythmic activity and significant increase in the baseline (p < 0.05; dashed black line). n = 27 cells for control, 24 cells for ORM-10103 10 μM and 10 cells for mepivacaine 50 μM + ORM-10103 10 μM

To test the hypothesis that NCX would contribute to the change of Ca^2+^ baseline in presence of mepivacaine, independent set of isolated cardiomyocytes from 3 mice were used to test two different NCX blockers alone and in combination with mepivacaine: NiCl_2_ (1 mM) and ORM-10103 (10 μM). In comparison to control solution (control 313.7 ± 21.45 nM; *n* = 27 cells), neither NiCl_2_ (252.5 ± 33.87 nM, *n* = 31 cells) nor ORM-10103 (361.5 ± 30.06; *n* = 24 cells) alone did not alter the baseline signal. Similar result was obtained with the peak area parameter, where NiCl_2_ (372 ± 78.56 nM*s) and ORM-10103 (474.3 ± 57.67 nM*s) were not statistically different from control solution (415.4 ± 36.13 nM*s). The recorded of isolated cardiomyocytes for each combination of NiCl_2_ or ORM-10103 with mepivacaine induced two major responses (Fig. 4). Most of the isolated cardiomyocytes that still had a normal phenotype under the combination of NCX blocker and mepivacaine showed an intense fluorescent signal and did not responded to electrical stimulation, indicating an increase in the cytosolic Ca^2+^ concentration and thus suggesting cell death. Few cardiomyocytes responded to initial electrical stimulation (*n* = 10 cells each combination), but entered in arrhythmia or increased the fluorescent signal with after few stimulations (Fig. 4a and b). In comparison to control, there was a significant increase in the baseline Ca^2+^ concentration in cardiomyocytes recorded with the combination of mepivacaine and NiCl_2_ (Fig. 4a: 508.1 ± 74.68 nM; F_6,32_ = 0.2067; *p* < 0.01) or mepivacaine with ORM-10103 (Fig. 4b: 450.8 ± 72.44 nM; F_6,32_ = 0.2067; *p* < 0.05).

## Discussion

Using a combination of Ca^2+^ transient data recording technique and data analysis based on biophysical parameters we described a new possible role of mepivacaine observed in murine isolated cardiomyocyte’s Ca^2+^ homeostasis. This combination provides fine details of mepivacaine’s action, showing new effect on Ca^2+^ release and uptake during EC–coupling. Mepivacaine’s effect was first established finding the IC_50_ value to reduce the peak area of the recorded Ca^2+^ transients calculated as 50.85 ± 6.79 μM. To assess the clinical relevance of the present study, our findings need to be interpreted in correlation to experimental conditions, which differ from physiological conditions in vivo. Typically, the therapeutic plasma levels found of mepivacaine range from 4 to 6 μg per ml [9]. However, thresholds for onset of local anesthetics toxicity are subject to a wide inter-individual range [18, 19]. Toxicity has been reported with a peak mepivacaine plasma concentration of 5.1 μg per ml [20], but the critical threshold dose seems to be a concentration of 6,27 μg mepivacaine-hydrochloride per ml blood plasma, which leads to a concentration of mepivacaine of 25,48 μM [21, 22]. Moreover, all anesthetics bind to plasma proteins, which decrease the effective circulating concentration of these anesthetics. Therefore, the actual free plasma concentration of anesthetic available to bind to tissues will be a fraction of the total plasma concentration [23, 24]. Despite the large difference between the clinical anesthetic dosage and the concentration used in our study, we showed in isolated murine cardiomyocytes a new possible role of mepivacaine affecting directly EC-coupling, by reducing Ca^2+^ release. It is also relevant to note that the same concentration of mepivacaine was used in Park & Suh [9] and in our results, suggesting that in rodents mepivacaine might have a small potency as described for humans, needing a supra-clinical dosage in order to observe any effect. For instance, in mouse neuroblastoma N2A an IC_50_ of 74.2 μM mepivacaine showed higher concentration- and state-dependent effect on tonic blockade of voltage-gated I_Na_ at holding potentials of − 100 mV [25]. Other reports show that varying mepivacaine’s concentration from 21 μM to 81 μM is used for antinociceptive effects by sciatic nerve blockade in mice [26], 40 μM mepivacaine to induce significant reduction on I_Na_ in isolated murine cardiomyocytes [7] and a wide range from 2 mM to 32 mM mepivacaine to ameliorate HIV sensory neuropathy in rats [27]. Therefore, the concentration of mepivacaine used here goes in concordance with previous results using mice and rats, supporting the evidences of lower mepivacaine potency in rodents.

Using the IC_50_ of 50 μM mepivacaine to analyze the biophysical parameters, it was observed that mepivacaine was able to reduce the Ca^2+^ release, without major effect on Ca^2+^ uptake. It is well established that mepivacaine has negative chronotropic and inotropic effects, as well ventricular arrhythmias and decrease of QTc- interval [5, 6, 28]. In dogs, there is also a significant increase of T-wave’s area-under-curve, lengthening of QTU interval and enhancing slow wave as interval of U-wave following T-wave [28]. These alterations on mepivacaine-mediated cardiac performance is explained in the ventricular cardiomyocyte to mepivacaine-induced I_Na_ reduction effect [7], which, as seen with lidocaine, reduces intracellular Na^+^ concentration, reducing Na-Ca exchanger reverse activity (inward Ca^2+^) and thus the reducing intracellular Ca^2+^ concentration and consequently myocardial contractility [29]. It is well established that mepivacaine becomes active after cytosolic protonation, allowing it to bind to the alpha unit of the voltage-gated Na^+^ channel. This reverse binding and thus blocking process occurs only from the cytosolic side when the Na^+^ channel is open resulting in a reduction of [Na^+^]_i_ [30–34]. It is well-established in physiological and pathophysiological conditions that Na^+^-Ca^2+^ exchanger has its most favorable thermodynamic activity occurs during diastole, extruding Ca^2+^ [35–38]. Under physiological conditions, NCX operates in the forward mode, moving Na^+^ to cytosol and Ca^2+^ to extracellular milieu, which results in a reduced intracellular diastolic Ca^2+^ levels [39]. This would be the case set by mepivacaine, where the reduced [Na^+^]_i_ activates NCX forward mode in order to avoid arrhythmias, resulting in a reduced sarcoplasmic reticulum Ca^2+^ after few Ca^2+^ transients. In order to test this hypothesis, we combined the use of mepivacaine with either two NCX blockers: NiCl_2_ and ORM-10103 both reduce the NCX forward and reverse currents [40]. The alone effect of either NiCl_2_ or ORM-10103 did not produce a significant change in the baseline, neither in the peak area of the recorded Ca^2+^transients, supporting others and our previous published data [12, 40]. The combination of mepivacaine with either NiCl_2_ or ORM-10103 produced two results: few cardiomyocytes responded with few electrical stimulation, followed by consecutive Ca^2+^ transients without electrical stimulation, similar to arrhythmic activity. However, most of the cardiomyocytes that received both drugs and still presented the characteristic isolated cardiomyocyte phenotype did not respond to the electrical stimulation, showing in some of them an intense background fluorescence signal. These results suggested that blocking both Na^+^ and Ca^2+^ homeostasis simultaneously would lead to arrhythmogenic activity and probably leading to cell death. Therefore, in combination with our baseline results, these data support the suggested hypothesis, where mepivacaine’s action via blocking Na^+^ channels significantly reduced intracellular Ca^2+^ concentration during diastole by enhancing the NCX reverse mode activity. However, this result must be carefully taken due to the limitation of the technique.

The negative inotropic effects of mepivacaine in ventricular papillary muscles have been previously reported [8, 9]. Park and Suh [9] studied the effects of 50 μM mepivacaine in isolated guinea pig and rat right ventricular papillary muscles, resulting in approximately 40% depression of peak force, concluding that the direct myocardial depressant effect of mepivacaine is likely to be caused by reduced Ca^2+^ release from the sarcoplasmic reticulum. David and colleagues [8] compared the inotropic and lusitropic effects of lidocaine and mepivacaine on rat left ventricular papillary muscles, showing that mepivacaine only induced a negative inotropic effect when added as a bolus for the highest concentration (1 mM) and this effect was significantly more pronounced with lidocaine than with mepivacaine. Moreover, a negative lusitropic effect was observed during isotonic contractions only for mepivacaine suggesting a reduction of Ca^2+^ in the sarcoplasmic reticulum. However, the fact that the post-rest potentiation was not significantly modified by mepivacaine or by lidocaine, indicating that Ca^2+^ efflux from the sarcoplasmic reticulum was not significantly influenced. Regarding the effects of mepivacaine on Ca^2+^ release, the results of David and colleagues [8] were in conflict with those reported by Park and Suh [9], where the latter suggest an inhibitory effect on Ca^2+^ release that was not confirmed by David and colleagues. Since the potential mechanism of mepivacaine’s myocardial depressant effect was not completely cleared, we investigated the consequences of mepivacaine influence in the general aspects of the Ca^2+^ transients, the parameters peak area, duration and D_50_ were analyzed. As shown in Fig. 2c and Fig. 2d, mepivacaine decreased peak area and D_50_ significantly. The present results suggest that the reduced Ca^2+^ transients could be caused by the activity of Na^+^-Ca^2+^ exchanger, inwarding Na^+^ and extruding Ca^2+^, which would lead to reduced sarcoplasmic reticulum Ca^2+^ concentration; alternatively, mepivacaine would also altered kinetics of Ca^2+^ release and/or Ca^2+^ uptake in the sarcoplasmic reticulum by an unknown mechanism. The biophysical parameters analyzed related to Ca^2+^ release and Ca^2+^ uptake showed that mepivacaine significantly reduced all three parameters which evaluate the Ca^2+^ release: peak, slope and time to peak. All together, these parameters suggest that mepivacaine would reduce the amount of Ca^2+^ released into the cytosol and its rate of release and thus, the time to reach its maximum. Finally, we observed no effect of mepivacaine on cardiomyocytes’ Ca^2+^ uptake by analyzing the parameters tau and time to uptake. However, this no effect cannot completely be excluded due to mepivacaine’s major effect on the Ca^2+^ release without challenging cardiomyocytes’ Ca^2+^ uptake mechanisms.

## Conclusion

This is the first study that evaluated the direct effects of mepivacaine on Ca^2+^ transients in isolated, adult mouse ventricular myocytes. Our findings in isolated cardiomyocyte preparations provide evidences to explain that the negative inotropic effect of mepivacaine found previously in cardiac muscle is due to its modulating effects on Ca^2+^ regulation at cellular level, specifically on Ca^2+^ release.

## Data Availability

The data produced in this manuscript was deposited in public archive under DOI 10.6084/m9.figshare.7713074.v1.

## Abbreviations

D_50_: Time duration at 50% of the maximum.
d_V_/d_tmax_: Maximum slope of action potential during depolarization.
F_max_: Maximum fluorescence intensity.
F_min_: Minimum fluorescence intensity.
IC_50_: Inhibitory effect at 50% of the response.
TB-A: Transfer buffer A.
TB-B: Transfer buffer B.
